# The effectiveness of an anti-stigma intervention in a basic police officer training programme: a controlled study

**DOI:** 10.1186/1471-244X-14-55

**Published:** 2014-02-25

**Authors:** Lars Hansson, Urban Markström

**Affiliations:** 1 Department of Health Sciences, Lund University, Lund, Sweden; 2 Department of Social Work, Umeå University, Umeå, Sweden

**Keywords:** Anti-stigma intervention, Discrimination, Police officer

## Abstract

**Background:**

Stigma and discrimination are still prominent features of the life situation of persons with mental illness, adding to the burden of the illness, causing a lowered self-esteem, quality of life and affecting possibilities of adequate housing and work. It is also a major barrier to help seeking. The deinstitutionalization of mental health services has led to a significant increase in contacts between the police and persons with mental illness. It has been argued that police officers should be provided education and training to enable them to interact adaptively and with good outcomes with people with mental illness. The present study is investigating the effectiveness of an anti-stigma intervention in a basic police officer training programme at a university in Sweden.

**Methods:**

The study was performed as a controlled pre-post intervention study using a comparison group, and a 6-month follow-up of the intervention group. Attitudes, mental health literacy and intentional behaviour were assessed. Main analyses were made on an intention to treat basis using repeated measures ANOVA. A total of 120 participants at a basic police officer training programme were included.

**Results:**

Time by group analyses showed improvements in the intervention group in overall score of attitudes and regarding the subscale Open minded and pro integration, in intentional behaviour (willingness to work with) and in 4 out of 6 items assessing mental health literacy. At the 6-month follow-up the intervention group had, as compared to baseline, improved attitudes in both overall score and in two of the subscales. Intentional behaviour had also improved in terms of an increased willingness to live or work with a person with mental health problems. Mental health literacy had improved in 3 out of 6 items.

**Conclusions:**

The anti-stigma intervention proved to be effective in changing attitudes, mental health literacy and intentional behaviour. Improvements mainly endured at the 6-month follow-up. The intervention seems promising in facilitating encounters between the police and persons with mental illness. Further studies are needed to disentangle the relative effectiveness of the components of the intervention before further implementation.

## Background

Stigma and discrimination are still prominent features of the life situation of persons with mental illness, adding to the burden of living with a mental illness. The deinstitutionalization of mental health services and the development of community-based services have not fundamentally changed this situation. Negative attitudes, stereotypes and discrimination are still prevalent. In fact there is evidence that public attitudes have not changed during the last two decades, or even turned worse in the case of people with schizophrenia [1]. Stigma and discrimination in many ways affect people with a mental illness causing a lowered self-esteem and quality of life [2], and affecting possibilities of an adequate housing, work and financial situation in a negative way [3,4]. It is also a major barrier to help seeking [5] causing delays, dropout and non-adherence in treatment [6,7].

The deinstitutionalization and efforts to integrate people with mental illness in the community has led to the development of a variety of mental health services delivering treatment and rehabilitation in community-based services. One aspect of this process is a widespread notion that contacts between the police and persons with mental illness have significantly increased [8-10], which is, however, not anchored in systematic research but mainly stemming from police and judicial sources based on daily experiences. What is evident is that the police force may nowadays be viewed as a prominent gatekeeper to both the criminal justice and mental health service system. An extensive review of studies in the field showed that around 5% of all police encounters involved persons with mental illness, 30% have had the police involved in their care pathway, and around 15% of referrals to emergency inpatient services involved the police [11]. Other studies have suggested that 10% [12], or in a broader perspective even up to 20% [13] of police contacts with the public involve people with mental illness. This indicates that the police now are one of the major “frontline extensions of the mental health service system”. The reasons for this are complex. Firstly, the mere presence of more people with mental illness living in the community will tend to increase contacts. Secondly, the vulnerable life situation that people with mental illness living in the community may experience, including higher levels of homelessness, unemployment, and drug abuse may induce contacts with the police and judicial system. Further reasons may be the criminalization of people with mental illness and not appropriate community-based mental health services being offered, leaving people untreated or not receiving evidence-based treatment [14].

A few studies of perceived discrimination have shown that contacts with the police are viewed as a significant area of discrimination or being unfairly treated. The INDIGO study found that 17% of people with schizophrenia felt discriminated by the police [15] and 6% of participants with a major depressive disorder [16]. In a recent Swedish study including a rather broad spectrum of people with mental illness, 13% reported that they felt unfairly treated by the police during the preceding two –year period [17]. A follow-up study in connection to an ongoing large scale anti-stigma programme in the UK found that 16.4% experienced discrimination from the police at baseline, and that this figure decreased not significantly to 16.1% in the years 2008–2011 [18].

The high frequencies of encounters between people with mental illness and the police, and results from the discrimination studies performed, highlight attitudes and behavior of the police in these encounters. There are rather few studies focusing police officers’ attitudes towards people with mental illness. A Canadian study by Trovato [19] indicated that the police had a positive orientation towards people with mental illness although their behavior was more consistent with an authoritarian and socially restrictive view. Another Canadian study [14] found that the police showed attitudes that were at least as benevolent as those of the general public. A study from Greece [20] showed more negative attitudes, where high percentages of the police perceived people with mental illness as dangerous, in need of continuous hospitalization, and unable to recover. An interesting vignette study from the USA [21] showed that police officers viewed persons with schizophrenia as being less responsible, more dangerous and more worthy of help than persons with identical vignettes provided, except for the information concerning mental illness. Another vignette study by Watson [8] showed that police officers were less likely to take action based on information provided by victims and witnesses with a mental illness, but no difference was found in response to suspects with or without a mental illness.

There are thus several reasons that police officers should be provided education and training in order to enable them to interact adaptively and with good outcomes with people with mental illness. Areas for education and training that have been stressed are: effective communication skills, understanding mental illness and its consequences, treating people with compassion and respect, and non-violent conflict resolution skills [11]. One attempt to resolve this issue has been the development and implementation of crisis intervention teams (CIT). CITs are proposed to improve the police officers ability to interact effectively, respectfully and safely with people with mental illness, and are based on police officers given a 40 hour specialized training [22]. Several studies, however none with a controlled design, have shown that CIT training results in improvement in attitudes and knowledge about mental illness, and improvement in confidence in identifying and responding to persons with mental illness [23]. Another study of a Canadian training programme with similar content but more stressing in vivo role-play training showed no changes in attitudes but significant positive changes in directly measured behaviour [24].

Results from studies of police attitudes and perceived discrimination from the police also call for a need to educate and train the police in an anti-stigma perspective. This research field has not gained much attention. A German study showed that a programme including seminars involving personal contacts between police officers and patients, relatives and health professionals, resulted in a decrease in assessed social distance and an amelioration in stereotypes concerning dangerousness and treatability of people with mental illness [25]. An evaluation of an educational intervention with the police force in England, also relying on personal contact with persons with own experience of mental illness, showed an improvement in attitudes four weeks after the final training session. There was also a report of increased willingness to work with a person with mental illness [26].

The present study is to our knowledge the first controlled study investigating the effectiveness of an anti-stigma intervention focusing the police force. It was performed in a basic police officer training programme at a university in Sweden, and the research questions were whether the intervention improved attitudes, mental health literacy and increased willingness to interact with people with mental illness after the intervention and at a 6-month follow-up.

## Methods and participants

### Design

The study was designed as a controlled intervention study using an intervention group and a comparison group. The study was performed at the basic training programme for police officers at Umeå University in Sweden. The intervention was delivered to police officer students as an add-on to a course dealing with psychiatry and mental health issues during their third semester of the training programme. The comparison group consisted of police students at the same university attending their first semester of the training programme. Data collection consisted of questionnaires covering knowledge, attitudes and intentional behavior towards mental illness and people with mental illness. In addition a short questionnaire investigating the participants’ familiarity with mental illness was used. Age, sex and civil status of the participants were also collected. Data was collected at the start and termination of the intervention and at corresponding time points in the comparison group. The intervention lasted for three weeks. A 6-month follow-up was performed in the intervention group. Based on the fact that the primary analyses of changes pre-post for the intervention period revealed no significant changes in any scales or items in the comparison group, the 6-month follow-up was not pursued in the comparison group.

### The intervention

The intervention was integrated in a psychiatry course and aimed at improving knowledge, behavior and attitudes towards people with mental illness. It was developed in cooperation with an ongoing Swedish national anti-stigma and anti-discrimination programme “Hjärnkoll” and had the following components: 1. an introductory lecture on attitudes towards people with mental illness, including a video presentation made by the national anti-stigma programme and focusing mental illness and attitudes. Actors in the video were people with lived experience telling their story (2 h), 2. two lectures by people with lived experience of mental illness covering schizophrenia and bipolar disorder respectively. These lectures mainly contained personal experiences of living with a mental illness, including struggles to overcome the illness and consequences for life and family situation (2 h), 3. six videotapes containing authentic cases casted by people with lived experience of mental illness shown as part of the lectures of the regular psychiatry course (3 h). The themes of these videos were psychosis, anxiety disorder, depression, bipolar disorder, suicide, and children in families with a parent with mental illness, (4 h). The two lecturers with lived-experience of mental illness also acted as counselors in practical in vivo training modules, giving their perspective and advice on how to best act and respond in these encounters. In these modules the police officer students had to respond to and take action in work-related situations involving persons with a mental illness. The latter was in these training situation played by professional actors (4 h). This intervention package was added to and integrated in a psychiatry course containing lectures on community mental health (3 h), lectures on various mental illnesses (8 h), legislation regulating the mental health area (3 h), and a case study presented at a seminar (2 h). The course in total lasted for around three full time weeks.

### Procedures and participants

Participants were recruited from the police officer training programme, in their first or third semester respectively. The course administrator approached the students, informed about the study and asked for informed consent. The students were also given written information about the study and were informed about anonymity and procedures of the data collection. The students in the comparison group were given additional oral information in order to secure that they were aware of the content of their participation in the study. No monetary compensation was offered the participants. In order to secure anonymity each student chose a unique personal code to be used on sealed envelopes used for delivering responses on the questionnaires at each data collection point. The intervention group consisted of police students in semester 3, with 60 eligible students, of which 54 consented to participate. Forty-six of these students participated in the post intervention and 6-month follow-up data collection. The comparison group consisted of students in semester 1 and included 72 students, of which 66 consented to participate and 59 of these participated in the post intervention data collection. No data was collected for those who chose not to participate.

### Measures

#### Community Attitudes towards Mental Illness (CAMI)

CAMI is a questionnaire originally developed by Taylor and Dear [27] which originally consisted of 40 items covering attitudes towards mental illness and people with mental illness. In the current study a Swedish version was used including 20 items categorized into three factors: “Open minded and pro-integration”, “Fear and avoidance” and “Community mental health ideology” The Swedish version has been tested regarded psychometric properties [28]. The response format of the questionnaire is a 6-point Likert scale ranging from 1 = do not agree to 6 = fully agree. The responses to some items were reversed in adding up scale scores so that higher scale scores indicate less stigmatizing attitudes. In the present study the internal consistency of the scale was found to be acceptable. Cronbach’s alpha was for the total scale = .82, Open minded and pro-integration = .73, Fear and avoidance = .72 and Community mental health ideology = .81.

#### Mental Health Knowledge Scale (MAKS)

MAKS comprises 12 items. Six items are related to stigma-related mental health literacy areas such as help seeking, ability to give advice, support, employment, treatment, and recovery, and 6 items inquire about knowledge of mental illness diagnoses. Items are responded to on a 5-point scale ranging from 1 = totally disagree to 5 = strongly agree. Higher scores indicate a better mental health literacy. The questionnaire has shown good psychometric properties with regard to reliability and validity [29]. MAKS was translated into Swedish and back translated into English according to standard procedures. The part of the questionnaire related to diagnoses was not used in the present study.

#### Reported and Intended Behaviour Scale (RIBS)

The RIBS is about reported and intended behavior in four different contexts: living with, working with, living nearby someone with a mental health problem, and continuing a relationship with a friend who gets mental health problems. There are eight items and the first four items to assess the prevalence of behaviour in the four contexts, while the next four items ask about intended future behavior within the same contexts. The response format of the first four items is Yes/No, and for the last four items a 5-point response scale is used ranging from strongly disagree = 1 to strongly agree = 5. A higher score in these four items indicate a greater willingness to interact with people with mental illness in these respects. The RIBS has been tested and found to be a feasible and psychometrically robust measure for assessing mental health-related reported and intended behaviour [30]. RIBS was translated into Swedish and back translated into English according to standard procedures. Only the four items referring to future intended behavior were used in the present study.

#### Background characteristics and familiarity with mental illness

Information about the participants’ age, gender and family situation (living alone or with partner) was also collected. Familiarity with mental illness was assessed by gathering information on whether the respondent was familiar with mental illness by own experience, by contact with friends, or by relatives with mental illness. Information on former education or work experience in the mental health care field was also collected. The response format in these items was yes/no.

### Statistics

Cronbach’s alpha was used to investigate the internal consistency of the CAMI scale and subscales. Repeated measures ANOVA was used to test for differences in group by time analyses in the pre-post controlled study. Intention to treat analyses were performed in comparisons between the intervention and comparison group, and missing data was imputed by the multiple imputation procedure in IBM-SPSS statistical package. G-power was used to investigate post-hoc power of the study and, using alpha probability error = .05, power was in all analyses with significant differences between groups shown to be >.99. Paired *t*-test was used to test for differences between baseline and 6-month follow-up in the intervention group. Independent samples *t*-test was used to test for differences between groups in the case of continuous data, and Chi^2^-test to test for differences between proportions. Cohen’s D was used to calculate between groups and within-group effect sizes (ES), and ES 0.2-0.5 were considered small, ES 0.5-0.8 medium and ES 0.8 and above as large effect sizes. IBM-SPSS version 21 was used for all statistical analyses.

### Ethical considerations

The study was performed in accordance with Swedish research ethics laws and the Helsinki declaration on research ethics. The study was based on informed consent and all participants were orally and in information sheets informed of the aims of the study, confidentiality issues and their rights to withdraw from the study at any point of time of their participation.

## Results

The intervention and comparison group differed slightly but significantly with regard to age, 26 (4.9) versus 24 (3.4) years of age, p = .040. There was also a difference with regard to living situation, more participants living alone in the comparison group, 65% versus 46%, p = .038. There was no difference with regard to distribution of sex (40% versus 53% women). Seventy-two percent in the intervention group and 68% in the comparison group reported that they had earlier experience of mental illness (own, friends, relatives, education, work experience) with no significant difference between the groups.

There were no significant baseline differences in attitudes, mental health literacy or intended behaviour scales or items used in the analyses. The intention to treat analyses showed a number of significant changes pre-post intervention in favour of the intervention group, see Table 1. Regarding attitudes, the repeated measures ANOVA showed that the intervention group significantly improved attitudes in the total score of CAMI (ES = .37), and in the subscale Open minded and pro integration (ES = .39), as compared to participants in the comparison group. Participants in the intervention group were also post-intervention more willing to work with (ES = .35) a person with mental health problems. Regarding mental health literacy, knowledge improved in four out of the six items assessed. Participants in the intervention group to a higher degree post-intervention stated that they knew what advice to give a person with mental health problems in order to get professional help (ES = .74), and they also to a higher degree endorsed that medication (ES = .71) and psychotherapy (ES = .78) can be effective treatments. They also to a higher degree had the opinion that people with mental health problems can fully recover (ES = .48). Further analyses including the significant baseline group differences in age and proportion of participants living alone as covariates, showed in no instance any significant interaction with the results from the time by group analyses.

**Table 1:** Repeated measures ANOVA comparing intervention (N = 54) and control group (N = 66) pre and post intervention with regard to attitudes, intentional behaviour and mental health literacy

**Area of investigation**	**Group**	** Pre**		** Post**		**F**	**Sign.**	**Effect size**
**Attitudes (CAMI)**		**m**	**SD**	**M**	**SD**			
Overall score	Intervention	4.65	.62	4.84	.56	6.19	.026	.37
	Comparison	4.62	.54	4.59	.63			
Open minded and pro integration	Intervention	4.29	.80	4.59	.75	6.08	.026	.39
	Comparison	4.26	.77	4.25	.82			
**Intentional behaviour (RIBS)**								
I would be willing to work with someone with a mental health problem	Intervention	3.25	1.25	3.51	1.35	6.27	.023	.35
	Comparison	3.16	1.23	2.98	1.14			
**Mental health literacy (MAKS**)								
If a friend had a mental health problem. I know what advice to give them to get professional help	Intervention	3.40	1.01	4.05	.65	6.29	.021	.74
	Comparison	3.48	.89	3.50	.84			
Medication can be an effective treatment	Intervention	4.00	.67	4.39	.61	10.27	.002	.71
	Comparison	4.25	.75	4.12	.80			
Psychotherapy can be an effective treatment	Intervention	4.32	.55	4.62	.65	9.14	.005	.78
	Comparison	4.43	.66	4.24	.66			
People with severe mental health problems can fully recover	Intervention	3.32	1.07	3.74	1.16	5.72	.049	.48
	Comparison	3.69	.91	3.61	1.06			

Intention to treat analyses.

The intervention group was also assessed 6-months post-intervention. Analyses of changes between baseline and the 6-month follow-up showed significant positive changes in attitudes with regard to total attitude scores (ES = .68) and the subscales Community mental health ideology (ES = .46) and Open minded and pro integration (ES = .33), Table 2. At follow-up they were also more willing to live with (ES = .32) and work with (ES = .30) a person with mental health problems. Mental health literacy also improved insofar as they at follow-up felt more able to give an advice to get professional help (ES = .75), were more inclined to agree that medication can be an effective intervention (ES = .56), and that people with severe mental health problems can recover (ES = .39). In Table 2 we also included within-group effect sizes for the intervention group in pre-post comparisons. In most cases effect sizes were similar and in the same range, except for the total score of attitudes where ES was twice as high at follow-up compared to post intervention (ES .68 vs .32), indicating that intervention effects were enduring during follow-up.

**Table 2:** **Comparisons of attitudes, intentional behaviour and mental health literacy and baseline and a 6-month follow-up of the intervention group (N = 46), paired sample *t*-test**

**Area of investigation**	** Baseline**		** 6-month follow-up**		**t**	**Sign.**	**Effect size pre- post**	**Effect size pre - FUP**
**Attitudes (CAMI)**	**M**	**SD**	**M**	**SD**				
Total score	4.80	.65	5.29	.80	8.28	.001	.32	.68
Community ideology	5.15	.63	5.45	.67	5.99	.001	.16	.46
Open minded and pro integration	4.31	.80	4.58	.80	3.04	.004	.38	.33
**Intentional behaviour (RIBS)**								
I would be willing to live with someone with a mental health problem	2.76	1.06	3.15	1.40	2.24	.031	.18	.32
I would be willing to work with someone with a mental health problem	3.19	1.24	3.58	1.35	2.72	.010	.20	.30
**Mental health literacy (MAKS)**								
If a friend had a mental health problem I know what advice to give them to get professional help	3.45	.94	4.05	.66	3.81	.001	.78	.75
Medication can be an effective treatment	4.00	.68	4.36	.61	2.88	.006	.61	.56
People with severe mental health problems can fully recover	3.47	1.08	3.89	1.06	2.30	.027	.37	.39

Within-group effect sizes for Pre-Follow-up and Pre-post comparisons are also shown in the table.

## Discussion

The main findings of the present study were that the intervention group of police officer students post intervention had improved their attitudes toward people with mental illness, were more positive to interact in the future and had improved their mental health literacy, in comparison with police officer students in the non-intervention group. The psychiatry course, in which the intervention was embedded, traditionally consisted of lectures on etiology and diagnosis of mental illnesses, the historical development of mental health services and on the life situation of people with mental illness living in the community. Persons with lived experience of mental illness did not participate. A recent meta-analysis of studies on effective anti-stigma strategies pointed out that interventions including social and personal face to face contacts with people with lived experience have shown to be the most effective strategy in changing attitudes of the public [31]. Since the add-on to the psychiatry course was mainly based on involving people with lived experience in personal contacts and videos reporting of lived experience, this speaks in favour of that the improvements in attitudes, mental health literacy and intentional behaviour actually may have been caused by this intervention.

Of special interest was the result that the intervention did not only improved attitudes, but also intentional behaviour, insofar as participants in the intervention group after the intervention were more inclined to work together with people with mental health problems. Furthermore this finding was validated by the 6-month follow-up of the intervention group, with similar or higher effect sizes points to that the effects of the interventions in these respects were enduring. The most common finding in intervention studies is diminishing effects during follow-up periods. We have no firm evidence for this not being the case in the present study. One possible explanation would be that the intervention period was quite intensive and short-term and that some of the issues raised during the intervention needed time and saturation to be personally integrated, reflecting the fact that attitudes towards people with mental illness have been reluctant to change. A further possible explanation would be that the in vivo training, including people with lived experience as mentors and instructors, revealed new ways of meeting and interacting with clients which might have paved the way for a generalization effect further on in parallel situations in the training programme. It should also in this context be noted that the intervention was made in a region in Sweden where at the time an anti-stigma programme was running. It may be that the present intervention was an “eye-opener” which made the police students more amenable for messages and events of this programme.

The general aims of the present interventions and the Crisis Intervention Teams, widespread in the USA, are similar in terms of aiming at a reduction of mental illness stigma. However, CIT is an approach with trained police officers, given 40 hours of specialized training including information on deescalation techniques and education on mental illness. CIT-teams provide first-line response to calls involving a person with mental illness and act as liaisons to the mental health system [23]. The present intervention is focusing police students not trained officers, and aims at changing attitudes and behaviour on a more generic level, as part of training for general police work, not work in a specialized team.

The findings of the present study must be interpreted with caution. The design of the study was not a RCT where differences between the groups were the anti-stigma intervention only. Comparisons were rather made between police officers attending a psychiatry course with an anti-stigma related add-on intervention, and students not attending this course at all. Thus, the design of the study does not rule out that it may have been the psychiatry course and anti-stigma intervention as a package that may have caused these differences. One option would have been to use a RCT design randomizing students within semester delivering the psychiatry course and giving one group of the students the anti-stigma add on intervention. The drawback of this design would be a loss of power in the study and the high risk of spillover effects, since students were attending the same course and engaging in discussions and social contacts which would possible have led to a substantial underestimation of the true effects of the anti-stigma intervention. A solution not at hand, would have been to randomize at another and more relevant unit of analysis, for example between universities having police officer training programmes. On the other hand the psychiatry courses differ in content and scope between universities which would have introduced problems concerning interpretation on another level. In either case we underscore the importance of further testing of this intervention, introducing further controls and disentangling the relative effects of the regular psychiatry course and the anti-stigma intervention in order to verify the preliminary conclusion that this may be an effective intervention.

## Conclusions

In conclusion, encounters between the police and people with mental illness have most likely increased as part of the deinstitutionalization process of mental health services. These encounters often take place in acute phases of a mental illness, and the outcome may be of great importance for the further course of illness. The police force have in several studies been pointed to as an important source of perceived discrimination, which makes the development of anti-stigma interventions focusing the police being of importance in conjunction to other efforts to diminish the burden of stigma and discrimination on people with a mental illness. Studies investigating the effectiveness of such interventions are scarce and the present study is an example of an intervention that in the light of further studies needed, may be a road ahead and worthy of future implementation on a broader scale.

## Competing interests

None of the authors declare any competing interests.

## Authors’ contributions

LH designed the study, made the statistical analyses and drafted the manuscript. UM was responsible for implementation of the intervention and the data collection procedures, participated in making up the study design, coordination of the study, and helped to draft the manuscript. Both authors read and approved the final manuscript.

## Pre-publication history

The pre-publication history for this paper can be accessed here:

http://www.biomedcentral.com/1471-244X/14/55/prepub
